# Case report: complete pathologic response to neoadjuvant ensartinib in locally advanced, ALK-positive lung squamous cell carcinoma

**DOI:** 10.3389/fonc.2026.1766882

**Published:** 2026-05-25

**Authors:** Yingyu Yang, Junkai Zhang, Zhirun Xie, Sirui Zhang

**Affiliations:** 1 Department of Pulmonary Oncology, Zhongshan City People’s Hospital, Zhongshan, China; 2 Liaoning University of Traditional Chinese Medicine Xinglin College, Shenyang, China; 3 Beijing University of Chinese Medicine DongFang College, Cangzhou, China

**Keywords:** ALK, case report, ensartinib, lung cancer, lung squamous cell carcinoma

## Abstract

ALK-positive of anaplastic lymphoma kinase (ALK) is rare in lung squamous cell carcinoma (LSCC), with an incidence of less than 1%. The role of neoadjuvant treatment with ALK inhibitors remains understudied. Here, we report the first case of stage IIIB, ALK-positive LSCC that achieved complete pathologic responses (pCR) to neoadjuvant ensartinib. In the current study, EML4-ALK positivity was identified by next-generation sequencing in a case with locally SCC. Rapid partial response was observed after four weeks of ensartinib. The patient continued to receive 6 weeks of ensartinib followed by right upper lobectomy with complete pathological response. To the best of our knowledge, limited data are available regarding the role of ensartinib as a neoadjuvant therapy for ALK-positive, non-small cell lung cancer (NSCLC) patients. Our case provided evidence that locally advanced, ALK-positive LSCC could benefit from neoadjuvant ensartinib, with an impressive response and favorable safety. Our findings may also extend the indications for targeted therapy to the neoadjuvant setting in locally advanced, ALK-positive, resectable LSCC.

## Introduction

Neoadjuvant chemotherapy has led to the increased opportunity for surgical intervention and decreased recurrence for patients with stage IIIB, resectable non-small cell lung cancer (NSCLC) (1). Neoadjuvant treatment with targeted drugs was also reported in patients with NSCLC harboring epidermal growth factor receptor (EGFR) mutations (2). However, the role of anaplastic lymphoma kinase (ALK) inhibitors as neoadjuvant treatment is rarely studied. Concurrent chemoradiotherapy or neoadjuvant chemotherapy remains the cornerstone for the treatment of patients with locally advanced NSCLC. However, patients with ALK rearrangements tend to have a poorer prognosis due to limited response to chemotherapy and lack of efficacy from immune checkpoint inhibitors (3). Several ALK tyrosine kinase inhibitors (ALK-TKIs) have been developed and approved as first-line treatments for metastatic ALK-positive non-small cell lung cancer (NSCLC) with improved outcomes and remarkable intracranial activity (4–6). In contrast, only a few case reports utilized these agents as neoadjuvant therapy in II-IIIB, ALK-positive disease, by which the efficacy can hardly be established (7, 8).

We reported a case with ALK-positive, stage IIIB LSCC that experienced a rapid response after two weeks of ensartinib, and underwent R0 resection after eight weeks of ensartinib, followed by adjuvant ensartinib therapy.

## Case

A 65-year-old, non-smoking female was referred to our institute with the complaint of one-week chest pain and was found to have a thoracic mass. Enhanced computed tomography (CT) on May 9th, 2024, revealed a solid mass measuring up to 55.5 x 43.5mm in the right upper lobe of the lung, and multiple enlarged lymph nodes in the mediastinum (**Figure 1A**). No distant metastasis was detected by abdominal CT, brain magnetic resonance imaging (MRI), or bone scan. With suspicion of lung cancer, fiberoptic bronchoscopy was performed. The microscopical appearance was consistent with the histological features of squamous cell carcinoma (**Figures 2A, B**). Immunohistochemistry (IHC) study revealed positive staining for P40. Given the clinical, radiological, and histological findings, the diagnosis was lung squamous cell carcinoma (cT3N2M0 IIIB, AJCC 8th edition TNM staging). A broader NGS Panel (Borning Rock, Guangzhou, China) was also performed, which confirmed positive EML4-ALK, high PD-L1 expression (TPS 90%), high tumor mutational burden (TMB-H, 12.0 Muts/Mb), and low microsatellite instability (MSI-L). A multidisciplinary team reviewed the case, and neoadjuvant treatment with oral ensartinib 225mg once daily was administered.

**Figure 1:**
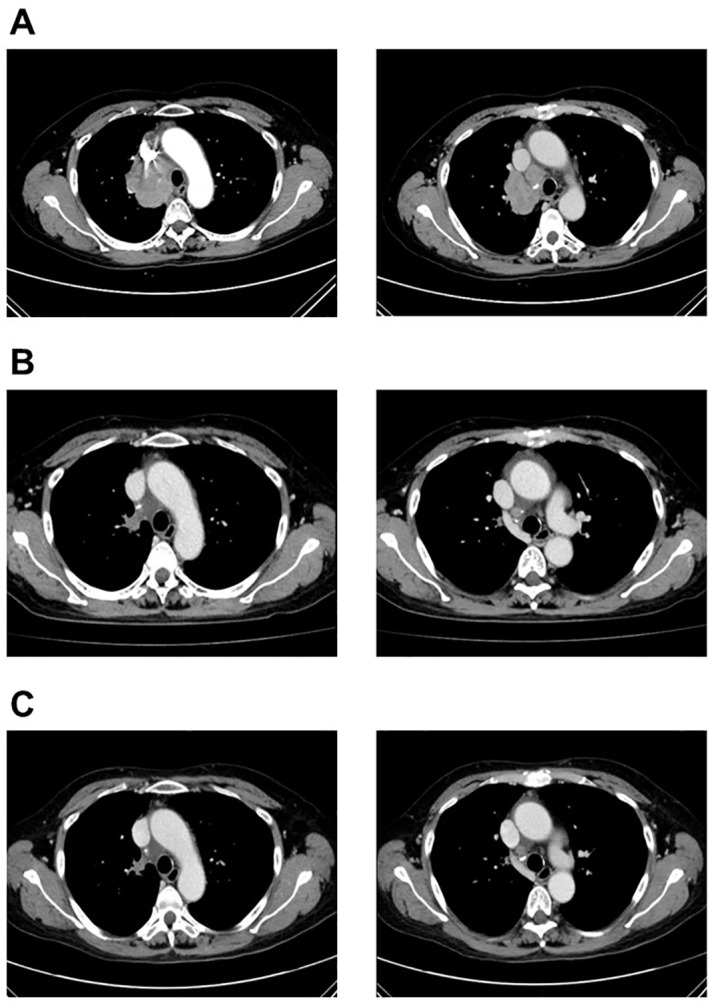
Response to neoadjuvant ensartinib in a patient with locally advanced, ALK-rearranged lung squamous cell carcinoma. **(A)** Squamous cell carcinoma of the right lung with metastasis in the mediastinal node was diagnosed in May 2024. **(B)** Downstaging from cT3N2M0 IIIB to a resectable status of cT1bN0M0 IA2 was confirmed by CT imaging after 8-week ensartinib. **(C)** CT scan performed before surgery showed a continuous partial response in the primary lesion and mediastinal node.

**Figure 2:**
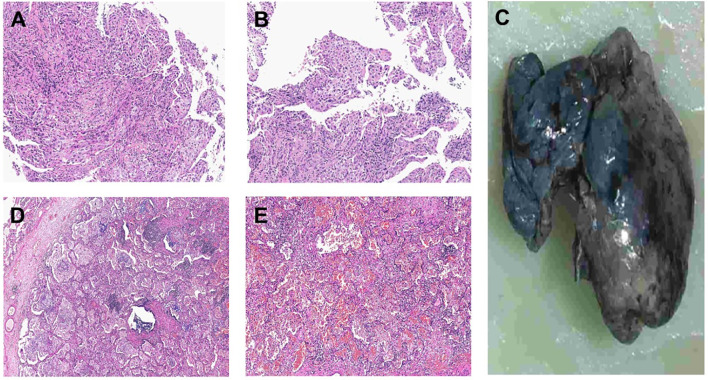
Complete pathologic response to neoadjuvant ensartinib **(A–E)**. **(A, B)** Trans-bronchial needle aspirate from right upper lobe lung mass showing lung squamous cell carcinoma at diagnosis. **(C)** Surgical resection showing a solid 3.2 cm fibrotic mass. **(D, E)** Hematoxylin and eosin staining revealed the tumor necrosis, stromal fibrosis, and inflammation with no residual viable tumor cells in the resected tumors.

After one month (May 31st, 2024), a remarkable tumor shrinkage of the lung nodule was observed, with a reduction of 37.5% (3.2 x 2.4cm). She continued to obtain a partial response in her lung lesion with the disappearance of mediastinal lymph nodes, and only mild rash was observed. After 8-week neoadjuvant ensartinib, repeated CT imaging confirmed downstaging from cT3N2M0 IIIB to a resectable status of cT1bN0M0 IA2 (**Figure 1B**). In July 2024 (**Figure 1C**), she underwent a right upper lobectomy with radical lymphadenectomy. Postoperative pathological examination revealed necrosis, inflammatory infiltration, fibrosis, and atypical hyperplasia of pulmonary epithelium in the resected lung lesions and lymph nodes without tumor cells, indicating a complete pathologic response (**Figures 2C–E**). After surgery, adjuvant ensartinib was offered, and there is no evidence of recurrence on imaging at 12 months post-operative follow-up.

## Discussion

Here we report an ALK-positive, stage IIIB, lung squamous cell carcinoma in a female who was treated with neoadjuvant ensartinib and subsequent surgery. She obtained a complete pathologic response without obvious toxicities except for mild rash.

ALK-positive is a well-established oncogenic driver that indicates sensitivity to ALK inhibitors. However, the role of neoadjuvant treatment with ALK inhibitors is still debated in patients with lung squamous cell carcinoma due to the limited evidence in the neoadjuvant setting and the rarity of ALK fusion in this population (less than 1%) (9). Our case achieved pathological complete response (pCR) after neoadjuvant ensartinib, providing important insights for this hard-to-treat patient population. Currently, neoadjuvant treatment for patients with ALK rearrangements remains understudied. Some retrospective studies have shown that ALK inhibitors are well-tolerated as neoadjuvant treatment in early-stage NSCLC with clinically meaningful pathological responses. However, evidence from prospective studies is still lacking. Targeted therapies have been widely used in the treatment of advanced or metastatic NSCLC, and findings from the ADAURA trial and the ALINA trial confirmed the efficacy and safety of adjuvant EGFR TKI or ALK TKI in early-stage NSCLC (10). Clinical trials are also investigating the role of targeted therapies as neoadjuvant therapy, primarily in stage IB-IIIA NSCLC patients harboring EGFR mutation. With respect to ALK-positive patients, its low incidence makes recruitment difficult for clinical trials. So far, there is no ALK TKI approved by the US FDA for the neoadjuvant treatment of early-stage NSCLC; only a few small studies and case series have described the efficacy and safety of ALK TKIs in this setting. In a 2019 study with 11 cases of ALK-positive, resectable locally advanced NSCLC that were treated with neoadjuvant crizotinib, 10 (91.0%) underwent an R0 resection, two patients achieved a pathological complete response, and three patients achieved downstaging of lymph nodes, which indicated that neoadjuvant crizotinib may be feasible and well tolerated in this setting (11). The phase II SAKULA trial evaluated the efficacy and safety of neoadjuvant ceritinib in patients with ALK-positive, resectable NSCLC. All patients (n=7) obtained ORR (100%), and six patients underwent surgical resection, in which five had R0 resection with major pathologic response (MPR) and pathological complete response (PCR) rate being 57% and 33%, respectively (7). NAUTIKA1 (NCT04302025) is an ongoing, phase II umbrella trial investigating the efficacy and safety of targeted therapies as neoadjuvant treatment in patients with resectable NSCLC. In the ALK-positive cohort, nine patients received neoadjuvant alectinib with a median treatment exposure of 8 weeks. Six out of nine patients obtained MPR, the MPR and CPR rates were 66.7% and 33.3%, respectively. Four (44.4%) and five (55.6%) patients had a partial response and stable disease, respectively; no patients had progressive disease during the neoadjuvant phase. Following neoadjuvant alectinib, eight patients had complete (R0) resection (8). One case report has reported a partial response to eight-week neoadjuvant crizotinib followed by surgical resection in a patient with ALK-positive stage IIIB lung adenocarcinoma. Post-operative pathological examination revealed less than 5% residual tumor cells in the primary tumor, with no evidence of lymph node metastasis (12). These findings have demonstrated favorable results of neoadjuvant treatment with ALK inhibitors. Despite the lack of data on the efficacy and safety in the neoadjuvant setting, ensartinib has shown an objective response rate (ORR) up to 75% in untreated, ALK-positive, metastasis NSCLC patients, indicating its potential benefit in the preoperative treatment of NSCLC (13). While the application of neoadjuvant treatment with other second-generation ALK-TKIs has been reported in case reports and studies with small sample size, emerging data has shown the clinical benefits of ensartinib in patients with resectable or locally advanced NSCLC. More recently, Lu and his colleagues reported the results from an interim analysis of the eXalt 3N study, in which neoadjuvant ensartinib resulted in an MPR of 70.6% and a pCR of 17.6% in patients with early-stage, ALK-positive NSCLC (14). All patients underwent R0 resection. Another multicenter, real-world study showed a major pathological response rate of 66.7% in ALK-positive NSCLC patients treated with enasrtinib (15). R0 resection was achieved in all patients regardless of whether they achieved pCR, indicating that the main function of neoadjuvant therapy is to facilitate resection. However, further studies are needed to investigate whether targeted adjuvant therapy is warranted after surgery.

Molecular pathology testing is crucial for the diagnosis and treatment of locally advanced NSCLC, with polymerase chain reaction (PCR), next-generation sequencing (NGS), fluorescence *in situ* hybridization (FISH), and immunohistochemistry (IHC) being commonly used in clinical practice. In this case, ALK positivity was determined by using PCR and NGS. With the current mainstream consensus and clinical research trend of “limited-duration treatment” strategy (approximately 2–4 treatment cycles that typically last 2 to 3 months), neoadjuvant therapy with ensartinib was then conducted. The duration of treatment is based on the rapid response of ALK-TKIs, including alectinib, crizotinib, and ensartinib. Furthermore, prolonged treatment duration (e.g., over 6 months) may increase potential resistance risk, accumulated toxicities, and delay surgery, which could raise the risk of tumor progression (though with low probability) or micro-metastasis.

Initially, cisplatin-based doublet chemotherapy was the standard of care for neoadjuvant treatment of resectable NSCLC, which provides a one-size-fits-all approach based on its cytotoxic effect. However, chemotherapy displayed limited efficacy in patients with actionable genomic alterations, and biologically driven treatment is recommended for patients with ALK-positive. The ORR occurred in 20-30% of patients after chemotherapy and 60-80% of patients after treatment with TKI in ALK-positive NSCLC, regardless of histological subtypes (4, 16). ALK-TKIs could become a new standard of care for resectable NSCLC with numerically higher MRP and pCR rates than chemotherapy, comparable to that of immune neoadjuvant therapy for NSCLC without actionable genomic alterations. The ALINA study has established the superior efficacy of alectinib over adjuvant chemotherapy in patients with ALK-positive NSCLC of stage IB to IIIA, by which alectinib was recommended as standard of care in the adjuvant setting. In a case series, one patient with ALK-positive, stage IIIB NSCLC achieved CR after receiving oral alectinib, she also achieved long-term survival (over seven years) without recurrence after lobectomy followed by adjuvant chemotherapy and alectinib (17). A 55.3% reduction in tumor size following alectinib treatment was reported in another case with ALK-positive, stage IIIA (cT1cN2M0) lung adenocarcinoma, which facilitated a salvage surgery (18). Despite metastases observed 20 months after surgery (alectinib was discontinued after surgery), reintroduction of alectinib achieved renewed disease control and a progression-free survival for 23 months. Taken together, these findings suggested that perioperative treatment with ALK-TKIs could provide a new paradigm for patients with ALK-positive NSCLC. Another advantage of neoadjuvant targeted therapy is the better tolerability compared with adjuvant chemotherapy. Although traditional chemotherapy is widely used as the backbone of treatment for various malignancies, ALK-TKIs are increasingly preferred owing to the improved efficacy and reduced toxicity. Furthermore, this case presents several unique biological features, including squamous histology, high PD-L1 expression, and tumor mutational burden despite ALK positivity. ALK fusion, which occurs almost exclusively in adenocarcinoma, was identified in our case of squamous cell carcinoma, in which a pCR was achieved after 8 weeks of neoadjuvant ensartinib. Patients with ALK rearrangements exhibited lower PD-L1expression levels and tumor mutational burden, which could be responsible for poor response to immune checkpoint inhibitors (3, 19, 20). Despite the promising efficacy of ensartinib in our case, the discrepancies between this case and previous studies should be considered, which limited the generalizability of our findings. Ensartinib resulted in a long-term clinical and radiological remission with no apparent side effects. This patient continued to receive adjuvant ensartinib and remained disease-free at 15 months after the surgery. Consistent with previous literature, this case added evidence supporting genetic alterations-based rather than histology-based triage for molecular testing and treatment selection. However, further investigation is warranted.

## Conclusion

Data is scarce regarding the application of neoadjuvant ensartinib in stage II-III disease, especially in those with squamous cell carcinoma. With a similar mechanism in adenocarcinoma, neoadjuvant/adjuvant ensartinib demonstrated favorable clinical benefits in squamous cell carcinomas carrying ALK fusions in the current case, with remarkable tumor shrinkage as well as reduced risk of recurrence, which ensured long-term benefits and offered the possibility of a cure, thereby providing a potential treatment option. However, caution should be taken when interpreting the results, as achieving pCR in stage IIIB lung squamous cell carcinoma with high PD-L1 expression and tumor mutational burden is relatively rare. Future studies are needed to provide evidence supporting the application of neoadjuvant ensartinib in ALK-positive, resectable NSCLC with longer follow-up.

## Data Availability

The datasets presented in this study can be found in online repositories. The names of the repository/repositories and accession number(s) can be found in the article/supplementary material.
